# Toe grip force of the dominant foot is associated with fall risk in community-dwelling older adults: a cross-sectional study

**DOI:** 10.1186/s13047-022-00548-1

**Published:** 2022-05-30

**Authors:** Satoshi Matsuno, Atsushi Yoshimura, Takuya Yoshiike, Sachiyo Morita, Yusuke Fujii, Motoyasu Honma, Yuji Ozeki, Kenichi Kuriyama

**Affiliations:** 1 Department of Psychiatry, Shiga University of Medical Science, Tsukinowa-cho, seta, Otsu, Shiga 520-2192 Japan; 2 Department of Physical Therapy, Osaka Yukioka College of Health Science, Osaka, Japan; 3 Department of Sleep-Wake Disorders, National Institute of Mental Health, National Center of Neurology and Psychiatry, Tokyo, Japan; 4 Cancer Center in Shiga Medical University Hospital, Otsu, Shiga Japan; 5 Department of Psychiatry, Ueno Hospital, Iga, Mie Japan; 6 Department of Physiology, Showa University School of Medicine, Tokyo, Japan

**Keywords:** Limb dominance; asymmetry; lower limb, Toe grip force, Dominant foot, Lower limb function asymmetry

## Abstract

**Background:**

It is unclear whether the toe grip force (TGF) of the dominant foot (DF) and the lower limb function asymmetry (LLFA) in older adults are associated with fall risk. Therefore, this study aimed to investigate the effect of lower limb properties (such as TGF, muscle strength, and plantar sensation) on the risk of falls in older adults, while considering the foot dominance and asymmetry of lower limb function.

**Methods:**

This study was a cross-sectional study. We determined whether the lower limb function of the DF and non-dominant foot (non-DF) and LLFA had any effect on the fall risk in 54 older adults (mean ± standard deviation: 72.2 ± 6.0, range: 60–87 years). We examined the participants’ fall history, Mini-Mental State Examination (MMSE) score, lower limb function, and LLFA. To determine fall risk factors, we performed logistic regression analysis, with presence or absence of falls as the dependent variable.

**Results:**

The independent variables were age, sex, MMSE score, two-point discrimination of the heel (non-DF) as plantar sensation index, and the TGF of both feet. Only the TGF of the DF was identified as a risk factor for falls (*p* < 0.05).

**Conclusions:**

In older adults, clinicians should focus on the TGF of the DF as a risk factor for falls.

**Trial registration:**

This study was retrospectively registered. https://center6.umin.ac.jp/cgi-bin/ctr/ctr_up_rec_f1.cgi.

**Supplementary Information:**

The online version contains supplementary material available at 10.1186/s13047-022-00548-1.

## Background

More than one-third of older adults aged ≥65 years experience falls in the United States (US) [1], and approximately 6% of older people who fall sustain bone fractures [2]. It has been reported that medical expenses for injuries caused by falls, which contribute to an increase in medical expenses in the US, exceed $31 billion [1]. In addition, the consequent frailty of bones after fracture in older adults imposes critical clinical and economic burdens on the society. Therefore, to maintain the health of older adults and reduce medical expenses, it is crucial to prevent falls among older adults.

Physical deterioration caused by aging-related factors, such as muscle weakness, sensory deficits, and balance dysfunction, is a well-known risk factor for falls [3, 4]. Toe grip force (TGF) affects balance and is a risk factor for falls in older adults [5–7]. Menz et al. [5] reported that plantar sensation and TGF contribute to the balance ability of older adults. However, to the best of our knowledge, no previous study has focused on limb dominance of the TGF.

Dominance is defined as a phenomenon in which one side of the left-right pair of body parts, such as the hands and feet, shows better results in cognitive and motor tasks than the other side [8, 9]. Yoshida et al. [10, 11] reported that the function of the dominant foot (DF) may affect standing posture control in dynamic balance. Based on these reports, it is necessary to pay attention to the function of the DF. A previous study [12] investigated the association between asymmetry of lower limb function and falls and found no significant association between the asymmetry of quadriceps femoris muscle strength (QFMS) and fall risk. In contrast, Koda et al. [13] reported that the asymmetry of TGF was associated with body sway while walking, suggesting the need to focus on the asymmetry of TGF. These studies suggest that for lower limb function, attention should be paid to the function of the DF and asymmetry of the lower limb function. However, the association of the TGF of the DF and the asymmetry of the TGF with fall risk remains unknown [5–7, 12]. Moreover, the effect of the properties of the non-dominant limb, such as muscle strength and plantar sensation rather than the TGF, has not been inclusively investigated. Therefore, the contribution of these properties to fall risk in older adults while considering foot dominance and asymmetry of lower limb function is also unknown.

This study aimed to investigate the effect of lower limb properties, such as TGF, muscle strength, and plantar sensation, on the risk of falls in older adults, while considering foot dominance and asymmetry of lower limb function.

## Methods

### Study setting

The study had a cross-sectional study design. All procedures were conducted at a laboratory at our university hospital. Participants underwent the entire process in a day. First, all the participants were provided information about the study and screened for the exclusion criteria. After obtaining informed consent, we assessed their eligibility using an original questionnaire to collect data on participants’ characteristics, Mini-Mental State Examination (MMSE), and plantar sensory function, QFMS, and TGF tests. All the procedures were primarily conducted by an experienced physical therapist with support from a clinical physician.

### Participants

A total of 54 community-dwelling older adults aged ≥60 years (25 men and 29 women) who responded to advertisements in a local library or hospital were enrolled in this study. All the participants lived independently in the local community and could visit our laboratory at the university hospital independently. Participants with current or previous paralysis, dizziness, body pain, limitations in range of motion and flexibility in body movements including the toe and forefoot, or sensory impairment and those with an MMSE score of ≤21 points were excluded from the study. Details on how the exclusion criteria were identified for participants in this study are reported in our previous study [14]. This study was conducted as a part of our previous study [14] and was approved by the Shiga Medical University Research Ethics Review Committee (reference number R2021–125). All participants provided written informed consent before participation in the study.

Participants who reported falling more than once in the past year were classified into the fall history group, and those who reported no fall history in the past year were classified into the non-fall group.

### Baseline measures

We examined basic characteristics of participants, such as age, sex, body mass index, DF, history of falls, and cognitive function. DF was identified as the foot used by participants to kick a ball [8, 9]. The participants were interviewed for the presence or absence of falls in the past year [15, 16]. History of falls was determined as “when a body part other than the foot touches the ground unconsciously” based on a previous study [17].

Cognitive dysfunction, which could also pose a risk for falls [3, 4], was evaluated using the MMSE. The MMSE is used internationally to assess the severity and progression of cognitive impairment with a maximum of 30 points for 11 items. The lower the score, the more severe the cognitive impairment [18].

### Physiological measures

**Fig. 1:**
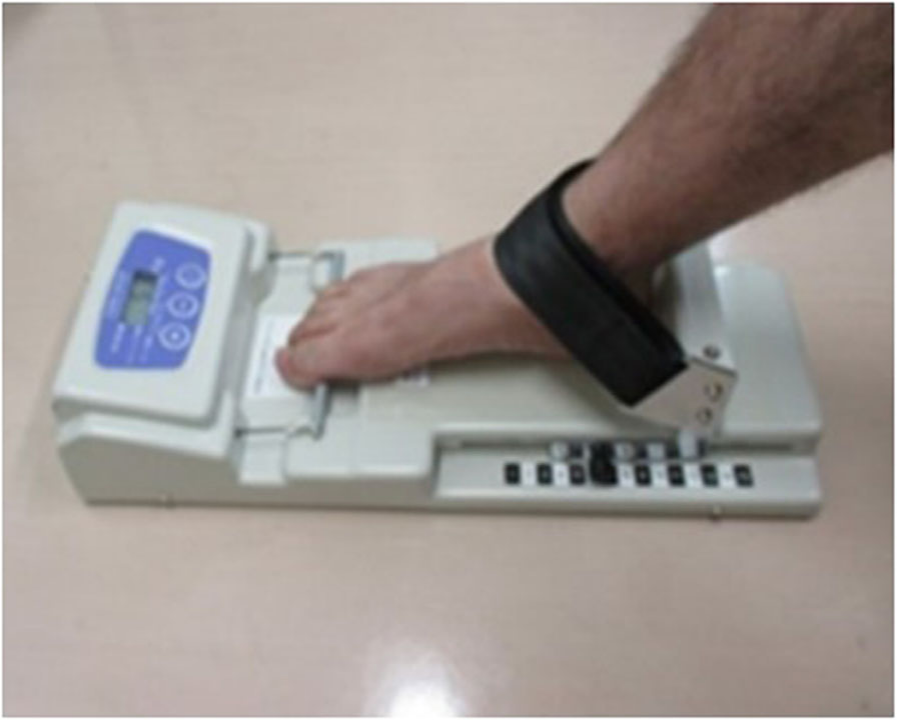
Evaluation of TGF using a toe muscle strength measuring device. To measure TGF, the participants sat in a chair barefoot. The participants then pulled a bar attached to the device using their toes, while their ankle and the sole of their foot were fixed to the device using a belt on the floor. We paid attention to the participants’ motion to ensure that their ankle and the sole of their foot were attached to the device and to prevent other compensatory movements while measuring the TGF. TGF: toe grip force

TGF and QFMS were evaluated to assess the lower limb muscle strength of the participants. TGF was measured using a toe muscle strength measuring device (T.K.K3364, Takei Scientific Instruments Corp., Osaka, Japan). To measure TGF, participants sat in a chair barefoot. The participants pulled a bar that was attached to the device using their toes, while their ankle and the sole of their foot were fixed to the device with a belt on the floor (Fig. 1). We paid attention to the participants’ motion to ensure that their ankle and the sole of their foot were attached to the device and to prevent other compensatory movements while measuring the TGF. TGF was calculated by dividing the measured value (kg) by the weight (kg) of the participants and was expressed as a percentage. It was measured twice for the DF and non-dominant foot (non-DF), and the average of the two measurements for each foot was included in the analysis. QFMS was measured using a handheld dynamometer (μTas F-1, Anima Corp., Tokyo, Japan). Participants sat in a chair with their sole lifted off the floor; we attached the device to the distal part of the lower leg and fixed it with a belt and then measured the isometric knee extension muscle strength. To calculate QFMS, the lower leg length (the distance from the knee joint space to the lateral malleolus) of the participants was measured. QFMS was divided by the participant’s body weight (kg) after multiplying the measured value (N) by the lower leg length (m). QFMS was also measured twice each for the DF and non-DF and then averaged.

**Fig. 2:**
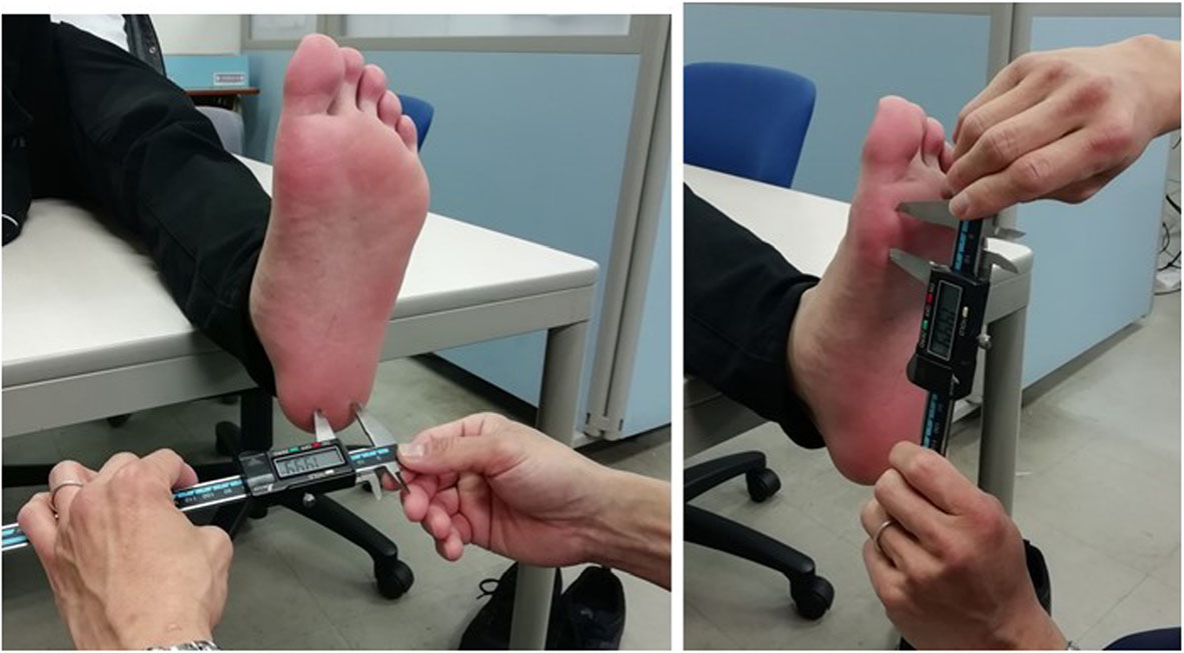
Evaluation of TPDS using a digital caliper. A digital caliper was used to measure TPDS of both soles of the participants’ feet. To measure TPDS, the participants closed their eyes, the soles of their feet were then brought into contact with the two points of the digital caliper, and the minimum distance between the two points of the calipers at which the participants could identify the two points distinctly was measured. TPDS: two-point discrimination sense

The two-point discrimination sense (TPDS) of the sole was measured as a somatosensory function. A digital caliper (AD-5765A-150, A&D Corp., Tokyo, Japan) was used to measure TPDS of both soles of the participants (Fig. 2). To measure TPDS, the participants closed their eyes, their soles were brought into contact with two points of the digital caliper, and the minimum distance between the two points of the calipers at which the participants could identify the two points was measured. The two points—the thenar and heel areas of the bilateral foot soles—were measured, and the average value of the two measurements was used as the adopted value for each sole.

To calculate the asymmetry of lower limb function, the asymmetries of TGF, QFMS, and TPDS were calculated using Eq. 1, as reported in a previous study by Carabello et al. [19].

1 $$ \mathrm{Asymmetry}=\left(\mathrm{weak}\ \mathrm{leg}\ \mathrm{function}\ \mathrm{value}-\mathrm{strong}\ \mathrm{leg}\ \mathrm{function}\ \mathrm{value}\right)/\mathrm{strong}\ \mathrm{leg}\ \mathrm{function}\ \mathrm{value}\times 100\%. $$

### Statistical analysis

Fisher’s exact test was used to compare sex, DF, and non-DF between the fall and non-fall groups. An unpaired *t*-test was used to compare continuous data between the fall and non-fall groups. To identify fall risk factors, we performed logistic regression analysis, with the presence or absence of falls as the dependent variable.

For independent variables, we entered variables with *p* values < 0.2 after the unpaired *t*-test and Fisher’s exact test according to the study by Coleman et al. [20]. Age and sex were entered into the logistic regression analysis model as independent variables regardless of the test results. For the logistic regression analysis, the presence or absence of a fall was considered the dependent variable, and age, sex, MMSE, heel TPDS (non-DF), and TGF of the DF and non-DF were considered the independent variables (model 1). Furthermore, to investigate whether lower limb function asymmetry was associated with fall risk, the presence or absence of a fall was considered the dependent variable, and age, sex, MMSE, and the asymmetry of TPDS, QFMS, and TGF were considered independent variables in another logistic regression analysis (model 2).

Statistical analysis was conducted using SPSS Statistics Ver. 27.0. (IBM Corp., Tokyo, Japan). Statistical significance was set as *p* < 0.05.

## Results

**Fig. 3:**
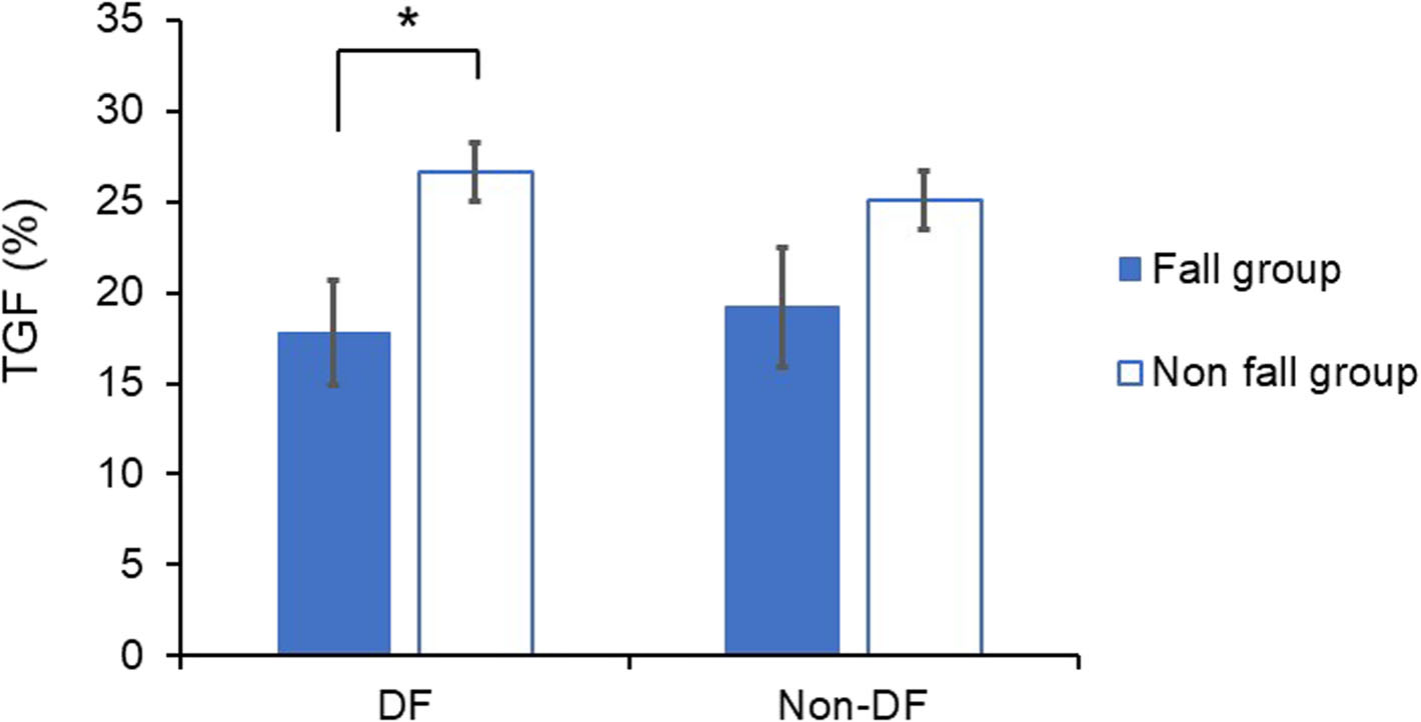
Differences in mean values of TGF between the fall and non-fall groups. Error bars indicate the standard error. TGF shows the percentage of measured TGF divided by the weight of participants. TGF: toe grip force, DF: dominant foot, non-DF: non-dominant foot. *Statistically significant difference: *p* < 0.05, determined using t-tests with equal variance

Table 1 shows the overall results of the fall and non-fall groups. Eight participants (14.8%) had a history of falls over the past year (Table 1). The overall proportion of participants with a right DF was 92.6%: 87.5% in the fall group and 93.5% in the non-fall group. Only the TGF of the DF showed a significantly lower value in the fall group than in the non-fall group (Table 1 and Fig. 3). There was no significant difference in the asymmetry of lower limb function between the fall and non-fall groups (Table 1).

**Table 1:** Characteristics and physical data of the participants (*n* = 54)

	Overall	Fall group	Non-fall group	
Variable	(*n* = 54)	(*n* = 8)	(*n* = 46)	*p*
Age, years	72.2 ± 6.0	73.6 ± 5.2	71.3 ± 5.9	0.307
Sex, men/women	25/29	3/5	22/24	0.441
DF, right/left	50/4	7/1	43/3	0.484
Height, cm	160.0 ± 9.2	159.0 ± 8.1	160.2 ± 9.4	0.742
Weight, kg	57.7 ± 10.3	54.2 ± 11.3	58.3 ± 10.1	0.306
BMI	22.4 ± 2.9	21.2 ± 2.8	22.6 ± 2.9	0.212
MMSE, score	28.8 ± 1.5	28.1 ± 2.0	28.9 ± 1.5	0.173
TPDS, mm				
TODF	16.9 ± 5.9	18.8 ± 5.4	16.5 ± 6.0	0.320
HODF	22.1 ± 8.9	24.8 ± 11.1	21.6 ± 8.5	0.349
TONDF	17.2 ± 5.2	15.8 ± 4.0	17.5 ± 5.4	0.419
HONDF	23.0 ± 9.0	27.7 ± 7.2	22.2 ± 9.1	0.115
Thenar asymmetry	18.7 ± 23.1	26.8 ± 35.7	17.3 ± 20.4	0.291
Heel asymmetry	26.6 ± 39.3	28.6 ± 29.0	26.2 ± 41.1	0.874
QFMS, Nm/kg				
DF	1.30 ± 0.36	1.25 ± 0.48	1.30 ± 0.34	0.714
Non-DF	1.21 ± 0.34	1.13 ± 0.38	1.22 ± 0.34	0.511
Asymmetry	−11.2 ± 8.6	−12.3 ± 5.2	− 11.0 ± 9.1	0.687
TGF, percentage weight ratio; %				
DF	25.3 ± 11.0	17.8 ± 8.2	26.6 ± 10.9	0.033
Non-DF	24.2 ± 10.5	19.2 ± 9.2	25.1 ± 10.5	0.142
Asymmetry	−14.1 ± 11.5	−16.0 ± 11.8	13.8 ± 11.5	0.621

Data indicate mean ± standard deviation

*BMI* body mass index, *MMSE* Mini-Mental State Examination, *TPDS* two-point discrimination sensory, *TODF* thenar of dominant foot, *HODF* heel of dominant foot, *TONDF* thenar of non-dominant foot, *HONDF* heel of non-dominant foot, *QFMS* quadriceps femoris muscle strength, *DF* dominant foot, *Non-DF* non-dominant foot, *TGF* toe grip force

The logistic regression analysis for model 1 suggested that only the lower value of the TGF of the DF (odds ratio, 1.293; *p* < 0.05; 95% confidence interval, 1.030–1.624) was associated with falls (Table 2). There was no significant association between the TGF of the non-DF and falls. The logistic regression analysis for model 2 suggested that there was no significant association between all independent variables and falls (Table 2).

**Table 2:** Multiple logistic regression analysis based on characteristics and physical data of the participants (*n* = 54)

	Model 1					
				95% CI		
Independent variable	*p*	SRCβ	OR	Lower	Upper	VIF
Age	0.814	−0.020	0.980	0.827	1.161	1.388
Sex	0.451	−0.804	0.448	0.055	3.618	1.232
MMSE	0.208	0.369	1.446	0.814	2.570	1.129
TPDS (HONDF)	0.054	−0.118	0.089	0.788	1.002	1.157
TGF (DF)	0.027	0.257	1.293	1.030	1.624	5.415
TGF (Non-DF)	0.118	−0.164	0.849	0.691	1.042	5.316
	Model 2					
				95% CI		
Independent variable	*p*	SRCβ	OR	Lower	Upper	VIF
Age	0.378	−0.069	0.934	0.802	1.087	1.227
Sex	0.553	−0.528	0.590	0.103	3.376	1.130
MMSE	0.187	0.342	1.407	0.847	2.338	1.174
TPDS thenar asymmetry	0.295	−0.016	0.984	0.954	1.014	1.096
TPDS heel asymmetry	0.865	−0.002	0.998	0.978	1.019	1.113
QFMS asymmetry	0.992	−0.001	1.000	0.908	1.100	1.094
TGF asymmetry	0.289	0.041	1.042	0.966	1.125	1.187

*SRCβ* standardization regression coefficient, *OR* odds ratio, *95% CI* confidence interval, *VIF* variance inflation factor, *MMSE* Mini-Mental State Examination, *TPDS* two-point discrimination sensory, *HONDF* heel of non-dominant foot, *TGF* toe grip force, *DF* dominant foot, *Non-DF* non-dominant foot

Since the variance inflation factor was < 10 for all variables, we judged that there was no problem of multicollinearity.

## Discussion

This study aimed to determine whether the TGF, QFMS, and TPDS of the lower limb are risk factors of falls by considering their dominance and asymmetry in community-dwelling older adults. There was no significant difference in QFMS, TPDS, and asymmetry of lower limb function in the non-DF between the fall and non-fall groups. In contrast, only the TGF of the DF was significantly lower in the fall group than in the non-fall group. The logistic regression analysis with presence or absence of a fall as the dependent variable after controlling for the other possible confounders identified the TGF of the DF as a risk factor for falls.

The foot soles are generally the only body parts in contact with the ground. Previous studies have reported that TGF is independently involved in balance ability, and its deterioration poses a risk of falls in community-dwelling older adults [5, 6], which is consistent with our findings. More specifically, our study result indicates the importance of the TGF of the DF for preventing falls. Yoshida et al. [10, 11] reported that the DF may contribute to spatial perception in standing posture control and posture control mechanism in dynamic balance. Furthermore, Demura et al. [21] reported that, compared with the non-DF, the DF was superior in its ability to regulate muscle exertion. These findings suggest that the TGF of the DF predicts the risk of falls.

In our study, QFMS was not different between the fall and non-fall groups, and multiple logistic regression analysis revealed no association with fall risk. Previous study have reported no significant difference in QFMS between the fall and non-fall groups in community-dwelling older adults [22], which is consistent with our study findings, suggesting that the TGF of the DF reflects the risk of falls more than QFMS.

Decreased somatosensory function in the lower limb is associated with falls [4]. However, there was no difference in somatosensory function measured by the TPDS between the fall and non-fall groups in this study, and the multiple logistic regression analysis showed no association with fall risk. A previous study reported that somatosensory function declined with age, and reliance on vision also increased [23]. The participants in both the groups in this study may have used vision adequately to compensate for the decline in somatosensory function owing to aging.

In this study, lower limb function asymmetry was not associated with falls. A previous study has reported that there was no significant association between asymmetry of the QFMS and fall risk in community-dwelling older adults [12], which is consistent with our findings. In the present study, the asymmetry of the sole TPDS and TGF was also not associated with falls. These results suggest that asymmetry of lower limb function has less impact on fall risk, indicating that the TGF of the DF plays a more important role in fall risk than the asymmetry of lower limb function.

Our results also suggest that the TGF of the DF more sensitively reflects the risk of falls than QFMS, TPDS in the sole, and asymmetry of lower limb function and that strengthening the TGF of the DF could reduce the risk of falls in community-dwelling older adults. Although the Berg Balance Scale (BBS) is a well-established scale used to predict the risk of falls [24, 25], its utility was validated in patients with a relatively high risk of falling, such as patients with Parkinson’s disease and post-stroke syndrome, but not in healthy older adults who live independently. Some studies on healthy individuals evaluated the risk of falls based on the history of falls over the past year [15, 16]. Therefore, we consider it more appropriate to investigate the association with falls based on the actual fall history of the past year rather than on the BBS.

This study has several limitations. First, since this was a retrospective study, it was not possible to demonstrate a causal relationship. Thus, we will consider clarifying the causal relationship through longitudinal studies in the future. Second, because this study targeted community-dwelling older adults who lived independently, the results of this study may not be applicable to older adults who need long-term care in their daily lives. Third, since this was the retrospective evaluation of fall history, which may have introduced mnemonic biases in this study. Fourth, identification of the DF of the participants was based on the foot used to kick a ball. The Waterloo Footedness Questionnaire Revised [26] has been widely used to identify the DF; therefore, it could have been used to determine the DF of participants in this study. Finally, intrarater reliability was not confirmed in this study. Therefore, in the future, we will investigate intrarater reliability in each evaluation.

## Conclusions

Our study findings suggest that in community-dwelling older adults, it is important to focus on the TGF of the DF as a predictor of falls rather than on the QFMS, TPDS in the sole, and asymmetry of lower limb function. Strengthening the TGF of the DF could reduce the risk of falls in community-dwelling older adults.

## Supplementary Information

**Additional file 1.**

## Data Availability

All data generated or analyzed during this study are included in this published article [and its supplementary information files].

## Abbreviations

BBS: Berg Balance Scale
LLFA: Lower limb function asymmetry
QFMS: Quadriceps femoris muscle strength
DF: Dominant foot
MMSE: Mini-Mental State Examination
TGF: Toe grip force
TPDS: Two-point discrimination sense
